# Inflammatory Markers and Immune Response to Pneumococcal Vaccination in HIV-Positive and -Negative Adults

**DOI:** 10.1371/journal.pone.0150261

**Published:** 2016-03-01

**Authors:** Anita S. Iyer, Noor M. Khaskhely, David J. Leggat, Jennifer A. Ohtola, Jessica L. Saul-McBeth, Sadik A. Khuder, M. A. Julie Westerink

**Affiliations:** 1 Department of Medicine, University of Toledo, 3000 Arlington Avenue, Toledo, Ohio 43614, United States of America; 2 Department of Public Health, University of Toledo, 3000 Arlington Avenue, Toledo, Ohio 43614, United States of America; 3 Department of Medicine; Department of Infectious Diseases and Department of Microbiology and Immunology, 135 Rutledge Avenue, Charleston, South Carolina 29425, United States of America; INSERM U1094, University of Limoges School of Medicine, FRANCE

## Abstract

**Background:**

Members of the Tumor Necrosis Factor (TNF)-superfamily have speculated roles in the response against T-independent type II antigens (TI-II) including pneumococcal polysaccharides (PPS). Dysregulation in their expression is associated with an enhanced risk for pneumococcal disease in neonates but their expression in other high-risk populations including HIV-positive individuals remains to be elucidated.

**Objective:**

To investigate signals that contribute towards PPS-response and identify potential anomalies that may account for diminished serological response in HIV-positive individuals post Pneumovax (PPV23) immunization.

**Methods:**

Markers of inflammation, C-reactive protein (CRP), IL-6, sCD27 and sCD30, were assessed in HIV-positive and -negative individuals as potential predictors of PPV23 response. Serum levels of B cell activating factor (BAFF), transmembrane activator and calcium-modulator and cytophilin ligand interactor (TACI), B cell maturation antigen (BCMA) and B cell expression of BAFF-R, TACI, BCMA, CD40 and CD21 were assessed in total (unselected) and PPS23F (antigen)-specific B cells of PPV23 immunized HIV-positive and -negative individuals.

**Results:**

CRP, sCD27, sCD30 and BAFF were significantly elevated in the serum of HIV-positive individuals but did not adversely affect PPV23 response. Assessment of PPS-specific B cells revealed enhanced TACI and reduced BAFF-R expression compared to unselected B cells in HIV-positive and -negative individuals. Surface TACI was similar but soluble TACI was significantly lower in HIV-positive compared to HIV-negative individuals.

**Conclusion:**

Current studies highlight a potential role for TACI in PPV23 response based on its enhanced expression on PPS-specific B cells. Although surface levels of TACI were similar, diminished soluble TACI (sTACI) in HIV-positive compared to HIV-negative individuals could potentially decrease BAFF responsiveness and Ig response. A better understanding of the role of TNF receptors could contribute to the design of improved pneumococcal vaccines.

**Trial Registration:**

ClinicalTrials.gov NCT02515240

## Introduction

*Streptococcus pneumoniae* is the most common cause of bacterial pneumonia in HIV-positive individuals. Incidence of invasive pneumococcal disease (IPD) is significantly higher in HIV-positive (173/100,000) compared to HIV-negative adults (3/100,000) [1]. Despite widespread availability of highly anti-retroviral therapy (HAART), newly diagnosed and HAART-experienced individuals are at a ≥35-fold increased risk for pneumococcal disease [2]. Consequently, pneumococcal vaccination is recommended for HIV-positive individuals [1].

As protection is dependent on production of opsonic antibodies against capsular pneumococcal polysaccharides (PPS), both quantity and quality of B cells play a critical role in vaccine response [3]. The onset of HIV is, however, characterized by early and severe B cell dysfunction including hypergammaglobulinemia, polyclonal B cell activation, increased cell turnover and impaired responses to vaccines amongst other defects. Although HAART has been successful in correcting several HIV-associated B cell defects, perturbations including loss of IgM memory B cells remain irreversible [4–6]. Given these perturbations, efficacy of pneumococcal vaccine in this population has remained controversial [7].

Capsular pneumococcal polysaccharides (PPS) are T-independent type II (TI-II) antigens as they elicit antibody production without direct T cell contact. In contrast, T-dependent (TD) response requires T-cell recognition of MHC restricted antigens [8, 9]. Secondary signals that contribute towards TD-response are well established but those governing TI-II antigens (PPS) have remained elusive [8–10].

Several lines of evidence suggest a role for TNF receptors in TI-II responses. Two TNF ligands, BAFF and APRIL act on B cells through shared receptors. BAFF binds BAFF-R, BCMA and TACI while APRIL binds TACI and BCMA. Extensive studies have focused on these receptors in the context of tumor and autoimmunity [8, 11, 12]. However, their role in immune defense is less explored. BAFF was found to play a non-redundant role among TNF ligands in supporting B cell survival. BAFF-/- mice exhibit profound reduction in antibody production against TNP (2, 4, 6-Trinitrophenyl)-Ficoll, a prototypic TI-II antigen [13]. BCMA-/- mice do not show a defect in primary B cell responses but survival of long-lived plasma cells was impaired compared to wild type controls [14]. Increasing evidence also links TACI with antibody production during TI-II responses. TACI-/-mice exhibit significant defects in secretion of NP (TI-II)-specific IgG and IgM [15]. In support, humans with TACI mutations show defective switched-memory B cells and recurrent susceptibility to bacterial infections [16]. Decreased expression of TACI, BAFF-R and BCMA in neonatal B cells also coincides with an enhanced risk for IPD [17] but its expression in HIV-positive adults remains unknown.

The role of TNFs CD40 and CD40L in TI-II responses is conflicting. CD40 and CD40L deficient mice were able to mount normal response to TNP (TI-II antigen) [8,9]. However, antagonistic antibody against CD40L inhibited IgM and IgG response in PPS immunized mice [9]. The relevance of this receptor in PPS-response thus remains uncertain in humans.

In addition to TNFs, complement C3d and its receptor CD21 have been speculated to contribute in pneumococcal defense [8, 10]. Binding of C3d to CD21 reduces the activation threshold required for B cell activation [10]. C3d conjugated capsular polysaccharides exert enhanced immunogenicity in mice and cultured human B cells [8, 9]. Reduced CD21 expression on infant B cells coincides with an inability to respond to PPS and increased susceptibility to infection [17]. Diminished CD21 levels have also been reported in B cells of HIV-viremic individuals [18] but its expression in PPS-specific B cells remains to be elucidated.

In our previous studies, we showed significantly diminished PPS-specific serological response in newly diagnosed and HAART-experienced HIV-positive adults compared to HIV-negative individuals post-PPV23 immunization [19, 20]. The influence of chronic inflammation and potential TNF-associated dysregulation in PPV23 response however remains to be determined.

The goals of the current study were to determine the underlying mechanisms that account for diminished PPV23-response in HIV-positive individuals. We compared the levels of pro-inflammatory cytokines in HIV-positive and -negative volunteers and evaluated their potential use as prognostic markers of PPV23-response. Next, we compared serum TNFs with speculated roles in TI-II responses between HIV-positive and -negative individuals to assess if potential dysregulation accounts for diminished PPV23 response. Lastly, we extended our previous work on PPS-specific B cells and characterized B cell surface expression pattern of BAFF-R, BCMA, TACI, CD40 and CD21. To our knowledge, expression pattern of speculated TI-II signals in overall (unselected) and PPS-specific (antigen-selected) B cells have not been examined in humans post PPV-immunization. Investigating PPS-specific B lymphocytes using fluorescently labeled PPS offers the novel advantage of assessing rare antigen-specific population while minimizing potential cross-reactivity by indirect labeling methods.

## Materials and Methods

### Subjects

100 volunteers were recruited after informed consent in this University of Toledo Institutional Review Board (IRB 106410, 107017 and 108321) approved, non-randomized study. All the volunteers completed a consent form and a written questionnaire. The work was carried out in accordance with the Declaration of Helsinki.

IRB approval was initially granted on 04/14/2009. All work on this proposal was completed 07/31/2014. All the volunteers included in the current study were recruited between the 02/01/2012 to 06/26/2014 period. The clinical trial protocol has been included as supplementary information in the version that was submitted to and approved by the University of Toledo IRB committee before the trial began. We did not intend this to be a clinical trial initially, however as we performed the work it became clear the work would benefit by becoming a clinical trial. We confirm that all ongoing and related trials for this intervention are now registered. It is available on Clinical Trials.Gov, identifier number NCT02515240.

HIV-negative volunteers in Toledo area served as immunocompetent controls. HIV-positive individuals were recruited from the University of Toledo Medical Center. Volunteers were questioned for pre-existing co-morbidities and exclusion criteria including history of cancer, immunosuppressing conditions, pregnancy, and splenectomy.

**Fig 1:**
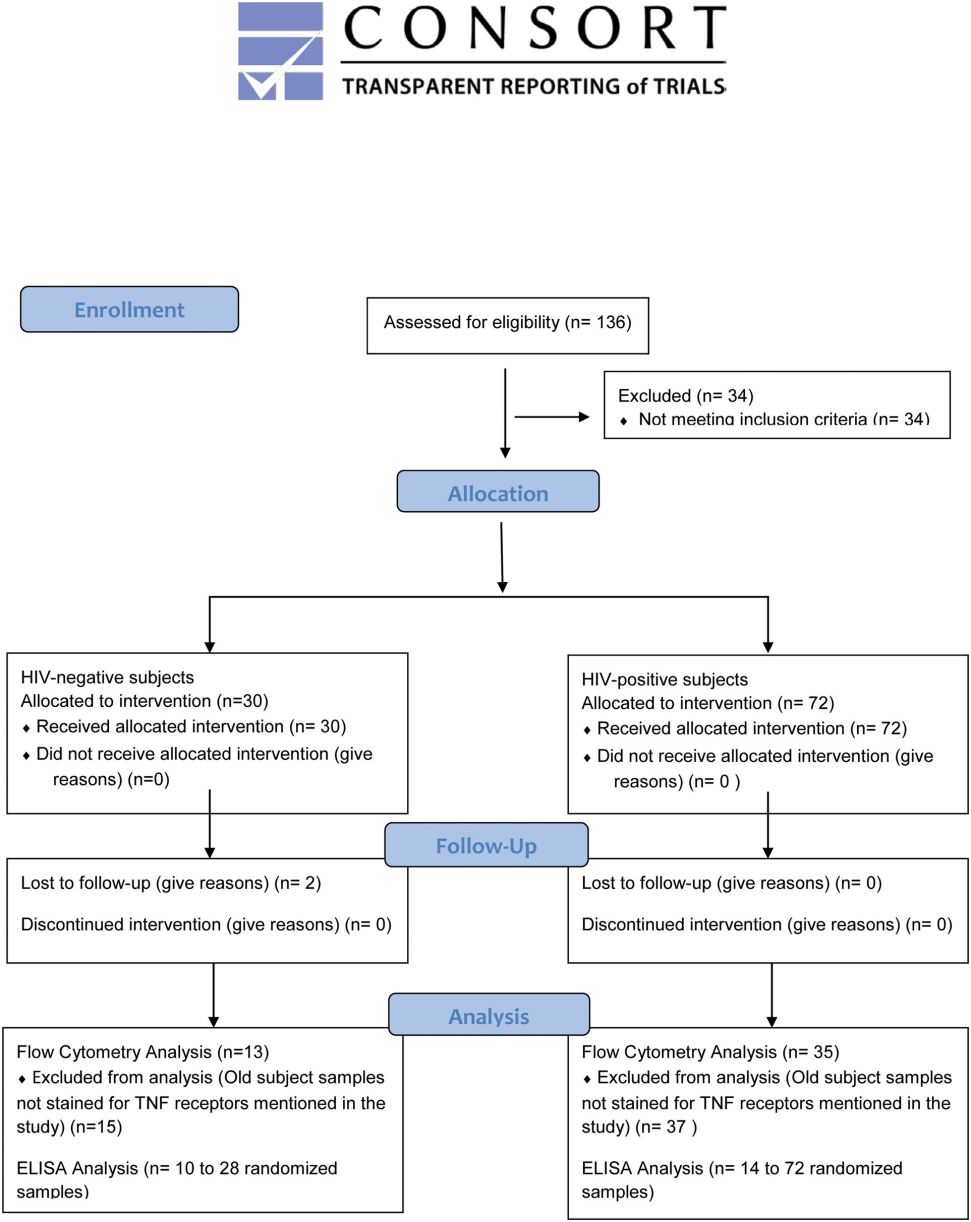
Consort Flow Diagram for Current study. 30 HIV-negative and 72 HIV-positive individuals were included in the study after informed consent. Out of this, 13 HIV-negative individuals and 35 HIV-positive individuals were included for inflammatory flow cytometric analysis based on the time of study initiation and sample availability. 10 to 28 randomized HIV-negative samples and 14 to 72 HIV-positive samples were used for inflammatory ELISAs.

Subject allocations and interventions are detailed in Fig 1.

Baseline characteristics of HIV-positive individuals are in Table 1. Volunteers were immunized with 0.5ml Pneumovax23 (Merck & CO., INC) intramuscularly at day 0 at the University of Toledo Medical Center. No adverse events were reported. Intra muscular pain at injection site lasting for up to 48 hours was a commonly reported side effect. Blood was drawn on day 0 (pre-vaccination) and follow up blood draw visits were on day 7 and day 30 post-vaccination.

**Table 1:** Baseline Characteristics of HIV-negative, HIV-positive HAART naïve and experienced individuals enrolled in the current study.

Parameters	HIV-negative	HIV-positive	
		HAART naive	HAART experienced
**Total n value**	**28**	**32**	**40**
**Mean Age (years)**	**29**	**30**	**49**
**Age Range**	**21–60**	**19–52**	**32–74**
**Sex, no. male/no. female**	**15/13**	**30/2**	**35/5**
**Race: White/Black/Hispanic/Asian**	**18/1/0/9**	**11/21/0/0**	**27/12/1/0**
**Viral Load (copies/ml)**	**N/A**	**464,200 ± 229,500 copies/ml**	**≤40 copies/ml**
**PPV vaccination**			
Primary Immunization	**Yes**	**Yes**	**Yes**
Secondary immunization (at the time of this study)	**No**	**No**	**Yes**
**CD4 count (cells/mm**^**3**^**) at the time of vaccination (Range)**	**N.D**	**2 to 918 cells/μl**	**69 to 1544 cells/μl**

N/A: Not applicable; N.D: Not determined

### Enzyme linked immunosorbent assay (ELISA)

Pre-vaccination (Day 0) serum samples were used to assess the baseline level of tested markers. BAFF (human BAFF Immunoassay; catalog SBLYSOB; R and D system), BCMA (human BCMA; catalog DY 193, DuoSet), TACI (TACI; catalog DY174; DuoSet), IL-6 (BMS213/2MST; eBioscience), CRP (BMS288INST; eBioscience), sCD27 (eBioScience; BMS286INST) and sCD30 (BMS240INST, eBioscience) ELISAs were performed based on manufacturer’s instructions. Samples were tested in duplicate for each enlisted marker.

PPS ELISAs were performed as previously described [21].

### Opsonophagocytic assay (OPA)

OPA was performed as previously described [19, 20] using day 0 and 30 volunteer serum samples.

### Labeling of polysaccharides

Conjugation of PPS23F to 5-(4,6-dichlorotriazinyl) aminofluorescein (5-DTAF; Sigma-Aldrich #36565) was carried out by Alamo Laboratories Inc, San Antonio, TX [22].

### Flow cytometry

Blood was draw on day 0 and 7 from enrolled volunteers for flow cytometry analysis. Lymphocytes were separated using Ficoll Hypaque method immediately and 1x10^6^ cells were added per staining tube for analysis the same day. Fluorescently conjugated pneumococcal PPS23F (5-DTAF) was added for the analysis of PPS-specific B cells [22]. Lymphocytes were also stained with anti-human CD19 (APC-Cy7), CD27 (PerCP-Cy5.5), IgM (APC) to assess unselected- and PPS-specific-naïve and -memory B cell subsets. Anti-human BAFF-R PE (eBioscience), BCMA-Alexa 700 (R&D System), TACI-PE (eBioscience), CD40-PeCy7 (eBioscience) and CD21-BV421 (BD) antibodies were used to assess expression pattern of receptors on unselected, PPS23F-specific total, memory and naïve B cell subsets. While adding stains to the tube, caution was taken to avoid fluorophor combinations that would cause spectral overlap.

Lymphocytes were plotted (FSC-A, FSC-H) for doublet discrimination. Singlet lymphocytes were assessed for the expression of CD19+ B cells. B cells were plotted using 5-DTAF: PPS23F to identify PPS23F-selected vs. unselected cells. PPS-selected and unselected cells were further divided into sub-populations: naive (CD27-IgM+/-), class-switched memory (CD27+IgM-) and IgM memory (CD27+IgM+) B cells. Total (unselected), PPS23F-selected and respective memory and naïve B cell subsets were analyzed for the expression of BAFF-R, TACI, BCMA, CD40 and CD21. All flow cytometry results were plotted and analyzed using fluorescence minus one controls (FMO).

Gating strategy for the inflammatory markers has been demonstrated in S1 Fig using BAFF-R as an example. TACI, CD40, BCMA, CD21 were evaluated similarly. 50,000 events were recorded. Percentages of each cell population within a group were then calculated and are reported as mean± standard error of mean (SEM).

### Statistical analyses

A pre-study statistical calculation indicated 20 volunteers per group as being sufficient for detecting upto 75% differences (alpha 0.05) with a power of 80% based on Fisher exacts test. We used Shapiro-Wilk's test to assess for normality. Independent t-tests were used to compare the differences in unselected and PPS-specific B cells within each group. Unpaired t-test and one-way analysis of variance (ANOVA) with Tukey’s Post Hoc test or Mann Whitney U test and Kruskal Wallis test was used to compare means of cytokine levels and B cell subsets across 2 and 3 donor groups respectively depending on variable normality. Correlations were assessed using Pearson’s and Spearman’s correlation test for normally and non-normally distributed data respectively. Data is presented as mean ± SEM and were analyzed using SPSS version 17.0 and Graph Pad Prism 5 software. p-values <0.05 were considered statistically significant.

## Results

### Pro-inflammatory cytokines are elevated in HIV-positive individuals but do not correlate with PPV23 response

**Fig 2:**
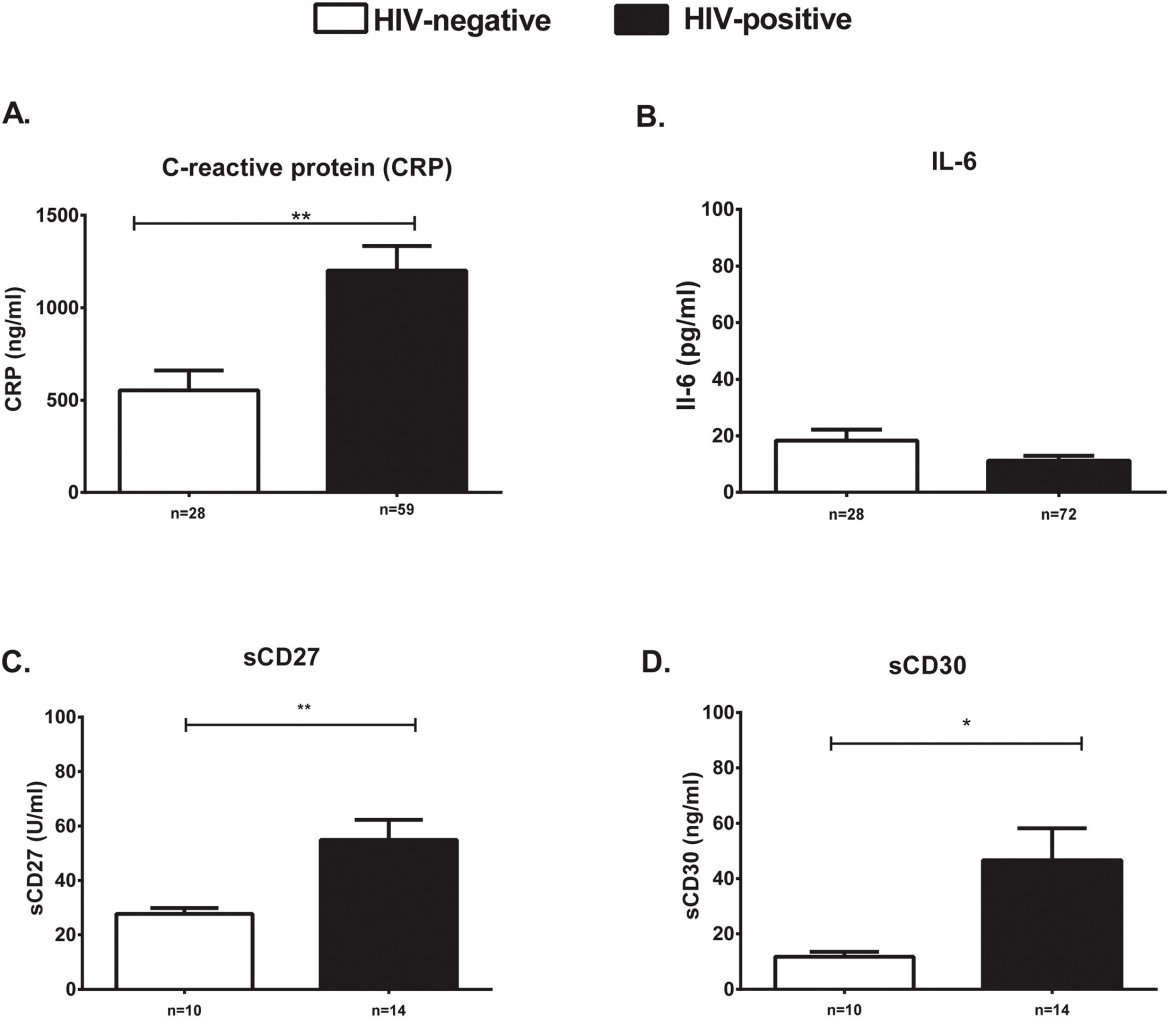
Pro-inflammatory markers are elevated in HIV-positive compared to HIV-negative individuals. Serum levels of CRP ng/ml (A), IL-6 pg/ml (B), sCD27 U/ml (C) and sCD30 ng/ml (D) were tested in HIV-positive (black bars) and -negative (white bars) individuals. Data is represented as mean ± SEM. *p<0.05, **p<0.01, ***p<0.001.

Recent studies highlight the inverse correlation between chronic expression of pro-inflammatory markers and impaired vaccine response [23–25]. We assessed serum levels of pro-inflammatory markers previously reported to be elevated in HIV-positive individuals as potential prognostic markers of PPV23 response. Elevated serum levels of CRP (554,300 ± 107100 pg/ml vs 1,246,000 ± 142,600 pg/ml), sCD27 (27.70 ± 2.176 U/ml vs 51.18 ± 5.353 U/ml) and sCD30 (11.80 ± 1.800ng/ml vs 52.73 ± 14.12 ng/ml) were noted in HIV-positive individuals (Fig 1). IL-6 was similar between HIV-positive and negative volunteers (18.32 ± 3.866 pg/ml vs 11.68 ± 1.969 pg/ml) (Fig 2).

An inverse but poor correlation was noted between CRP levels but not other markers and 23F-specific OPT response in HIV-positive individuals (r = -0.3, p = 0.01) (S2 Fig).

### Serum BAFF and TACI are differentially expressed in HIV-positive and negative individuals

**Fig 3:**
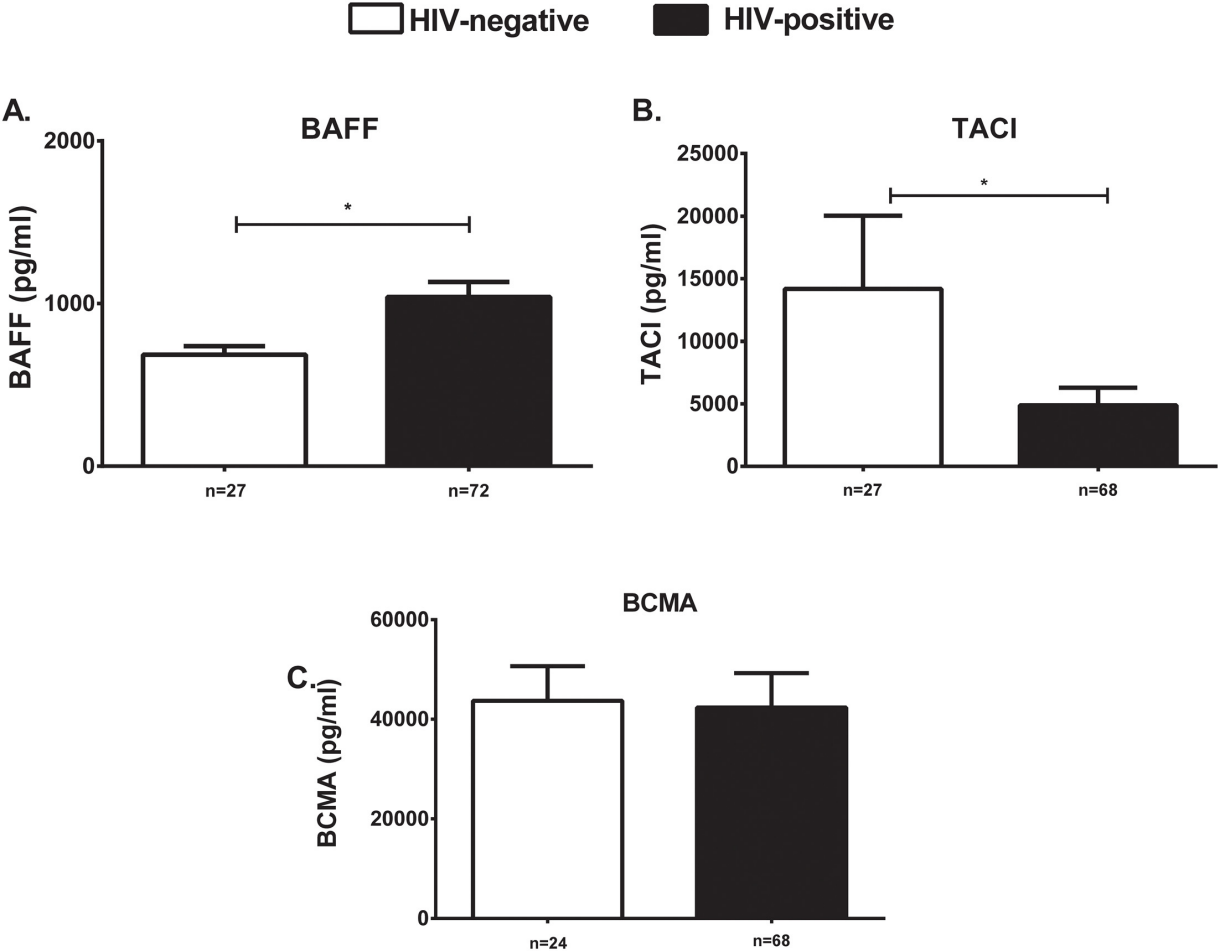
Serum BAFF and sTACI are dysregulated in HIV-positive individuals. Serum levels of soluble sBAFF pg/ml (A), sTACI pg/ml (B) and sBCMA pg/ml (C) were tested in HIV-positive (black bars) and -negative (white bars) individuals. Data is represented as mean ± SEM. *p<0.05, **p<0.01, ***p<0.001.

To identify mechanisms that may contribute towards diminished PPV23 response, we investigated serum levels of TNFs critical for B cell homeostasis. We noticed significantly elevated levels of serum BAFF in HIV-positive (1070 ± 106.9 pg/ml) compared to -negative individuals (686.8 ± 52.52 pg/ml) (Fig 3A). Conversely, sTACI was higher in HIV-negative (14180 ± 5867 pg/ml) compared to HIV-positive individuals (4820 ± 1481 pg/ml) (Fig 3B). sBCMA levels were not significantly different between HIV-positive (36550 ± 2583 pg/ml) and negative (43700 ± 6944 pg/ml) individuals (Fig 3C).

An inverse but poor correlation was noted between serum BAFF and 23F OPT (r = -0.4, p = 0.03) in HIV-negative individuals. No other correlations were found between serum levels of tested TNFs and post-vaccination OPT (S3 Fig).

### Unselected B cell expression of speculated TI-II signals is not different between HIV-positive and -negative individuals

**Fig 4:**
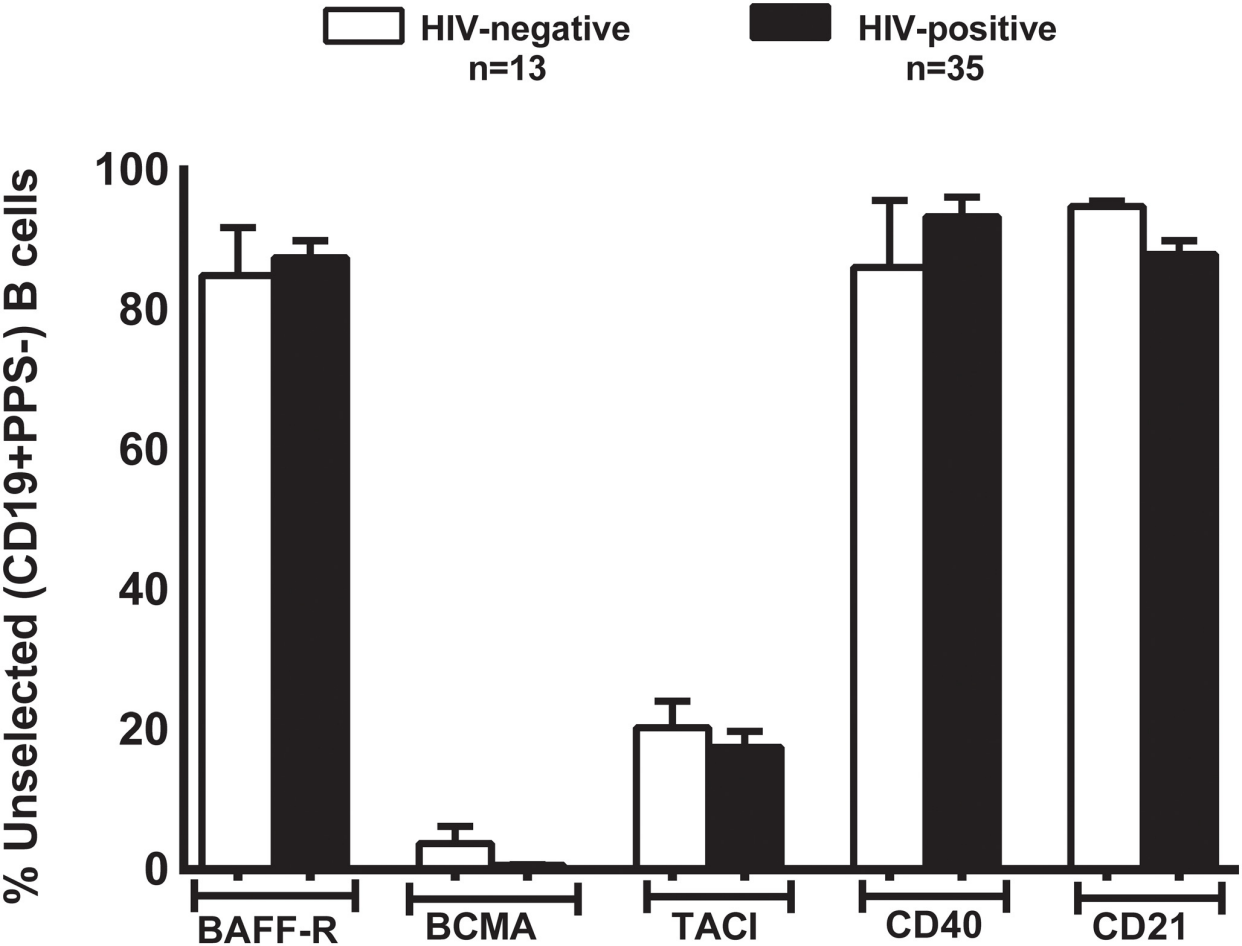
TNF and complement receptor CD21 expression is similar in the total B cells of HIV-positive and HIV-negative individuals. Expression of receptors BAFF-R, BCMA, TACI, CD40 and CD21 were analyzed in total B cells of HIV-positive (black bars) and -negative (white bars) individuals. Data is represented as mean ± SEM. *p<0.05, **p<0.01, ***p<0.001.

We compared unselected B cell expression of BAFF-R, BCMA, TACI, CD40 and CD21 in HIV-positive and -negative volunteers and did not find them to be significantly different (Fig 4). Unselected B cells expression of these receptors were similar on day 0 and 7 (data not shown). Day 7 has been shown as a representative.

### Expression of CD40 was significantly higher in PPS23F selected B cells of HIV-positive individuals

We next evaluated the surface expression pattern of BAFF-R, BCMA, TACI, CD40 and CD21 on PPS-specific (antigen-specific) B cells day 7 post-immunization. We chose day 7 as PPS-specific B cells peak in the peripheral blood during this time [22, 26]. We chose PPS23F as it is a vaccine serotype and its prior inclusion in our previous studies.

**Fig 5:**
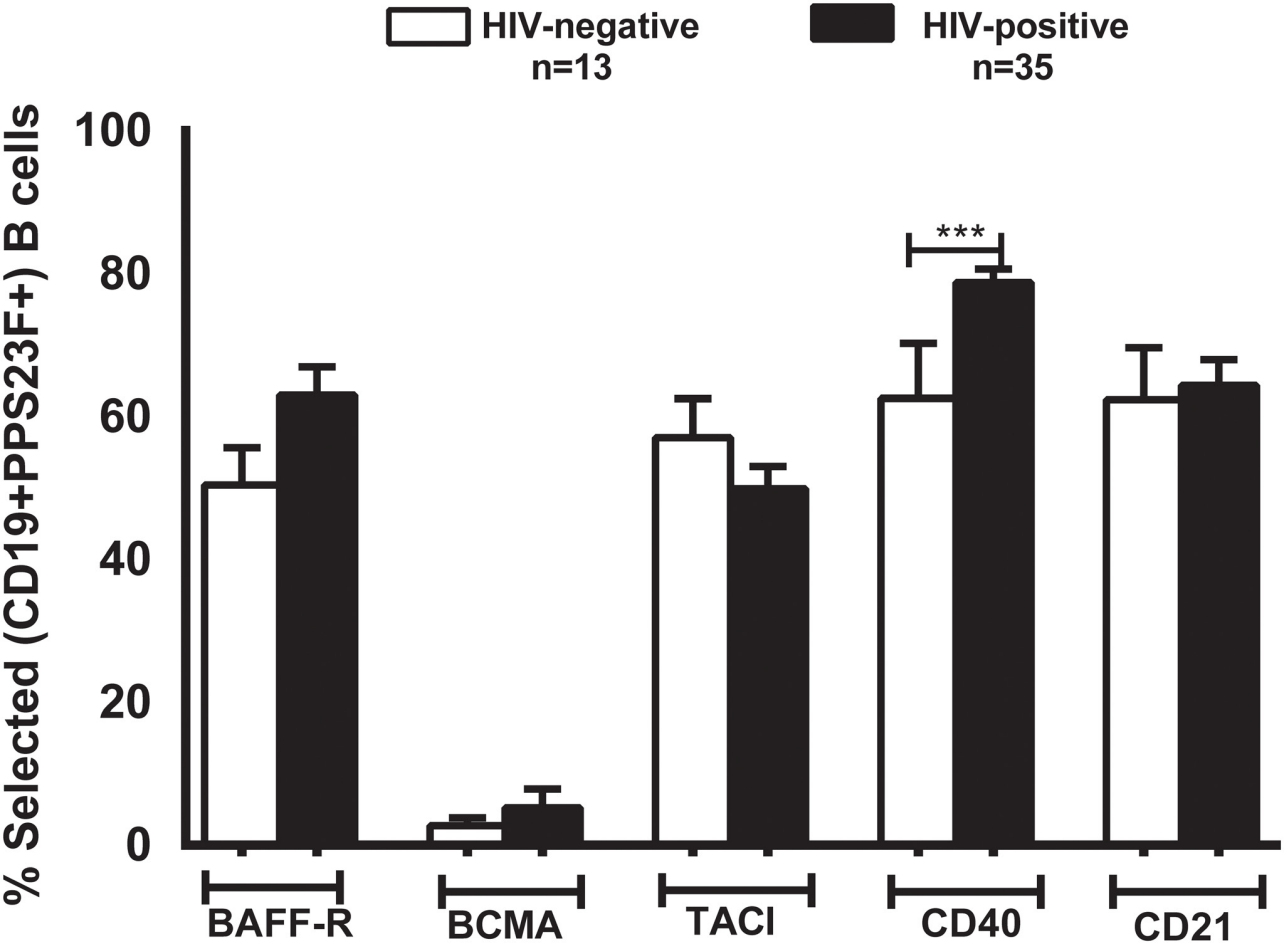
PPS23F-specific B cells showed enhanced expression of CD40 in HIV-positive compared to HIV-negative individuals. Post-vaccination (Day7) analysis of PPS-specific B cells against the tested serotype 23F showed similar levels of BAFF-R, BCMA, TACI, CD21 but higher levels of CD40 in HIV-positive (black bars) compared to HIV-negative (white bars) individuals. Data is represented as mean ± SEM. *p<0.05, **p<0.01, ***p<0.001.

Surface expression of CD40 was significantly elevated in HIV-positive individuals. No significant differences were noted in the expression of BAFF-R, BCMA, TACI and CD21 between HIV-positive and -negative individuals (Fig 5).

### PPS23F-specific memory B cells show higher expression of TACI compared to unselected memory B cells

We evaluated expression of BAFF-R, BCMA, TACI, CD40 and CD21 on unselected and PPS23F-selected memory (CD27+ IgM+/-) B cells.

**Fig 6:**
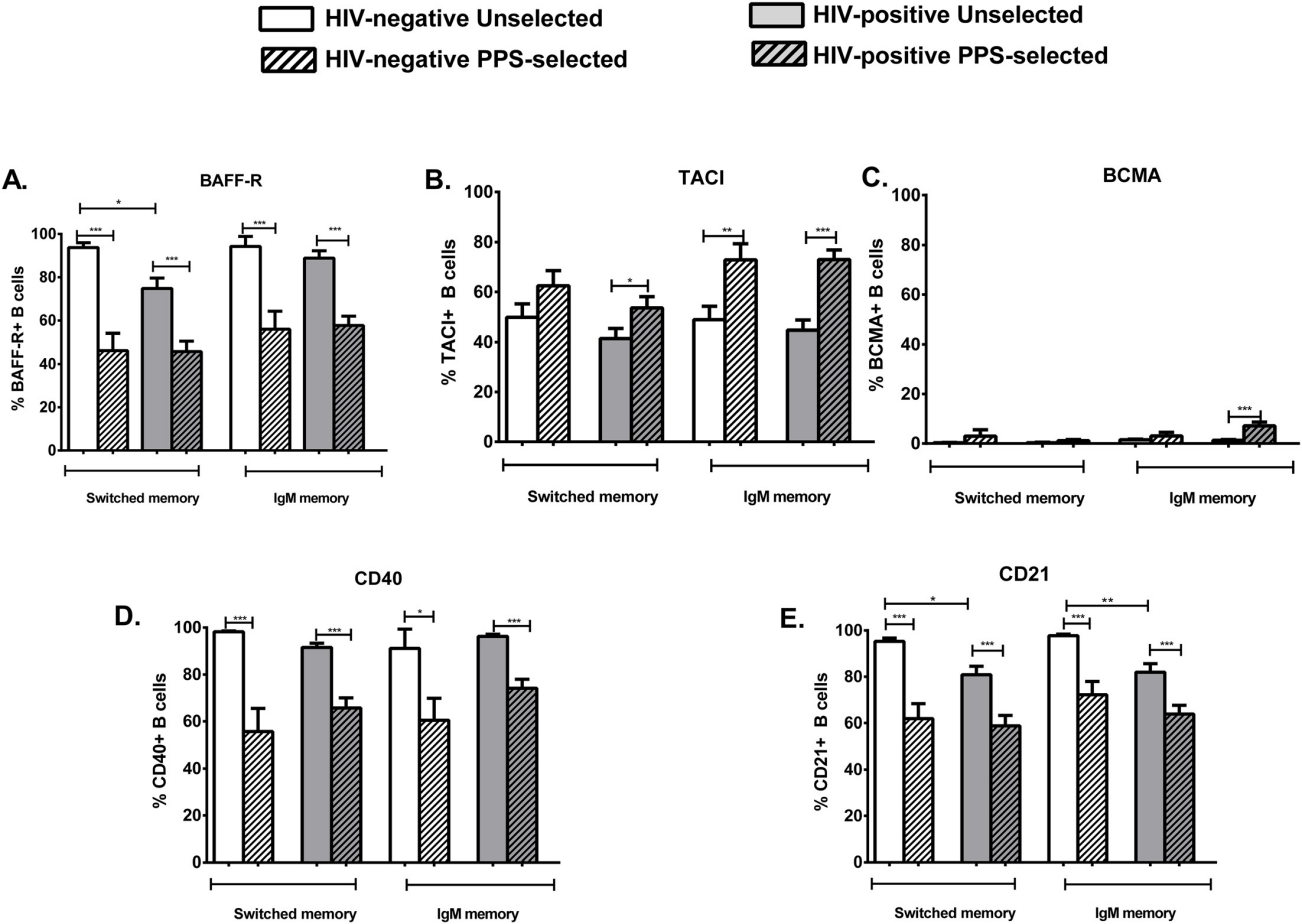
PPS23F-specific memory B cells showed enhanced expression of TACI in HIV-positive and HIV-negative individuals. Surface expression of BAFF-R (A), CD40 (D) and CD21 (E) on day 7 PPS23F-selected B cells of HIV-positive (grey bars with slanted stripes) and HIV-negative (white bars slanted stripes) individuals compared to day 0 unselected B cells in HIV-positive (plain grey bars) and HIV-negative (plain white bars) individuals. Data is represented as mean ± SEM. *p<0.05, **p<0.01, ***p<0.001.

PPS23F-specific memory B cells (both switched and IgM) in HIV-positive and -negative individuals showed reduced expression of BAFF-R, CD40, CD21 compared to respective unselected B cells. In contrast, increased TACI expression was noted in PPS-specific memory B cells compared to unselected memory B cells in HIV-positive and -negative individuals (Fig 6). With the exception of CD21, significantly lower in unselected memory B cells of HIV-positive individuals, no other differences were noted between HIV-positive and -negative volunteers.

PPS-selected and unselected naïve B cells expression of receptors is shown in S4 Fig.

### HIV-viremic individuals show enhanced serum BAFF, sCD30 and decreased B cell expression of CD21

Stratifying HIV-positive individuals based on the receipt of HAART revealed higher levels of BAFF and sCD30 in the serum of HAART naïve, HIV-viremic individuals (S5A and S5B Fig).

Similarly, B cell expression analysis revealed significantly lower percentages of CD21 expressing unselected -memory and -naïve B cells in HIV-viremic individuals (Fig 4C and 4D) consistent with other reports [4].

## Discussion

We previously reported significantly diminished serological response to PPV23 in newly-diagnosed and long-term HAART-experienced HIV-positive adults compared to HIV-negative individuals. These findings suggested significant damage to critical PPS-responding B cell subsets early on during HIV-infection that failed to recover with HAART, regardless of CD4+ T cell reconstitution[19, 20]. Consistently, we noted significantly diminished percentages of PPS-specific IgM memory B cells in HIV-positive individuals which correlated with diminished OPT indicating its pivotal role in pneumococcal defense[19, 20]. In our current studies, we further explored overall competence of total and PPS-responding B cells in HIV-positive and -negative volunteers by assessing receptors and cytokines speculated to be critical for PPS response. We grouped HAART-naive and HAART-experienced individuals as no significant differences were noted between them unless otherwise stated (S4 Fig). Additionally, no differences were noted in the expression of these molecules regardless of CD4 count (data not shown).

Reports in the recent years view HIV-infection as an accelerated aging process. Inflammaging, defined as excessive and continued presence of pro-inflammatory cytokines is a common feature in elderly, in people with HIV and is associated with morbidity and mortality [23, 24]. Increasing evidence links chronic inflammation with impaired vaccine responses in the elderly. High levels of serum TNF-α in the elderly was associated with impaired influenza vaccine response [25, 27]. Elevated levels of pro-inflammatory markers including members of TNF-family have been reported in the serum of HIV-infected individuals [28] although their relevance to pneumococcal vaccine response remains unknown. We tested pro-inflammatory markers previously reported to be elevated in the serum of HIV-positive patients [28] as potential prognostic markers for PPV response. Consistently [28], we noted significantly elevated levels of sCD27, sCD30 and CRP in HIV-positive volunteers. Normalization of sCD30 but not sCD27 and CRP in HAART-experienced individuals was consistent with literature [28–30]. Elevated levels of the tested serum markers did not correlate with quantitative and qualitative antibody levels indicating response to each vaccine is different and not always adversely influenced by generalized inflammatory status.

To further probe mechanisms that may contribute towards diminished PPS-response in HIV-infected individuals, we investigated serum levels of TNF ligands and its receptors with speculative roles in TI-II responses. Serum levels of ligand BAFF was significantly higher in HIV-positive individuals and could be a result of B cell hyperactivation during HIV-disease [4, 31]. Serum levels of BAFF receptor BCMA were similar but sTACI was significantly lower in HIV-positive compared to HIV-negative individuals. TACI renders B cells responsive to BAFF and APRIL mediated signals promoting class switching and TI-II responses. Although we did not assess the serum levels of these molecules over time, it is possible that antigen recognition by BCR triggers surface TACI cleavage to a greater degree in HIV-negative individuals promoting downstream signals and Ig response.

We evaluated surface expression of BAFF-R, BCMA, TACI, CD40 and CD21 in unselected (total) and PPS23F selected (antigen-specific) B cells to characterize the nature of cells that respond to PPS and identify potential anomalies in HIV-positive individuals. TACI and BAFF-R have implicated roles in TI-II responses [13, 15, 32]. While BAFF-R is expressed throughout B cell development except in plasma cells, TACI upregulation is known to occur only after B cell activation [33]. In support of this concept, B cells in influenza vaccine recipients exhibit decreases in BAFF-R+ B cells and increases in TACI+ B cells concomitant with antibody response [27]. In line, we noted lower expression of BAFF-R and higher expression of TACI in PPS-selected cells compared to -unselected B cells. Our findings are in line with reports that suggest antibody-secreting cells lose BAFF-R expression [27, 34, 35].

Surface TACI is particularly high in marginal zone (MZ) and B1 B cells [33], key B cell subsets in pneumococcal defense [36]. Consistently, loss of TACI impairs IgM response and survival in pneumococcus infected mice [37]. In humans, subsets of circulating CD27+IgM+ (IgM memory) B cells are viewed as splenic MZ B cells and B1 B cells with a defensive role against pneumococci [38, 39]. Consistently, we noted higher expression of TACI in PPS-specific CD27+IgM+ B cells compared to unselected IgM memory and other PPS-specific subsets in both HIV-positive and -negative individuals. These findings in PPS-specific B cells are consistent with reports that suggest an upregulation of TACI in antibody forming cells, highlighting a potential role for TACI in pneumococcal defense [27]. However, no differences in the surface levels of TACI were observed between HIV-positive and -negative volunteers.

Analysis of unselected compartment revealed the presence of CD21^lo^ IgM and switched memory B cells in HIV-positive, specifically HIV-viremic individuals consistent with literature [4]. Surprisingly however, CD21 expression on PPS-specific B cells were lower than unselected B cells and comparable between HIV-positive and negative volunteers. The levels of CD40 were lower on PPS-specific B cells as well compared to unselected B cells. Implications of reduced percentages of CD21 and CD40 on PPS-specific B cells remains unclear. A limitation of this study is that it’s a non randomized controlled trial.

To the best of our knowledge, this is the first comprehensive study that directly characterizes phenotypic expression of speculated TI-II signals on PPS-specific B cells. Direct characterization of PPS-specific B cells is critical as it highlights the nature of cells that respond to antigens. Based on our findings, PPS-specific B cells in both HIV-positive and -negative volunteers downregulate BAFF-R expression and enhance the expression of TACI indicating a role for TACI in PPV response. This is consistent with studies where TACI-/- mice were impaired in pneumococcal defense [37]. Additionally, enhancing TACI expression on marginal zone (MZ) B cells by addition of CpG enhanced Ig secretion in murine studies [32]. In support of this concept, HIV-infected individuals receiving CpG along with pneumococcal vaccine showed enhanced Ig response in a placebo controlled trial, although mechanisms behind this effect were not investigated [40]. In our study, surface TACI expression was not dyregulated, but its serum levels were low in HIV-positive volunteers. It is possible that BCR recognition of antigens cleaves surface TACI from activated B cells to a greater extent in HIV-negative compared to HIV-positive individuals. Cleaving TACI could then potentially trigger robust downstream signaling and Ig secretion by mechanisms that needs further characterization. In support of this concept, ectodomain shedding of TACI from activated B cells by proteases belonging to a disintegrin and metalloproteinase (ADAM) family was demonstrated in a recent study. The generated soluble form sTACI was analogous to its membrane form and bound ligands BAFF and APRIL [41]. Although sTACI was shown to have regulatory effects, its activation or Ig inducing function in our study cannot be dismissed given its well-established dual roles. How sTACI might impact vaccine response needs further understanding. Other receptors such as toll like receptors, could be defective in B cells of HIV-positive individuals leading to diminished PPV23 response and needs further elucidation. These studies are ongoing. Further investigation of PPS-specific B cells along with in-vitro stimulation studies may enlighten the mechanisms governing PPV23 response. In conclusion, our studies unravel the expression of TNFs and receptors speculated to be critical in PPV23 response in both healthy and high risk pneumococcal disease groups. Improved understanding of these cytokines, receptors and downstream signaling will be useful while considering the design of new robust pneumococcal vaccines.

## Supporting Information

S1 Fig Gating strategy to analyze unselected and PPS23F-specific B cells and subsets. Lymphocytes were stained with fluorescently labeled PPS23F and antibodies for phenotypic characterization of cells. Lymphocytes were plotted (FSC-A, FSC-H) for doublet discrimination. Singlet lymphocytes were assessed for the expression of CD19+ B cells. B cells were plotted using 5-DTAF: PPS23F to identify PPS23F-selected vs. unselected cells. PPS-selected and unselected cells were further divided into sub-populations: naive (CD27−IgM+/-), class-switched memory (CD27+IgM−) and IgM memory (CD27+IgM+) B cells. Total (unselected), PPS23F-selected and respective memory and naïve B cell subsets were analyzed for the expression of BAFF-R as indicated. All flow cytometry results were plotted and analyzed using fluorescence minus one controls (FMO). 50,000 events were recorded. Data are represented as mean ± SEM. *p<0.05, **p<0.01, ***p<0.001.
(TIF)

S2 Fig Serum levels of pro-inflammatory markers do not correlate with post-vaccination 23F opsonophagocytic titers (OPT). Serum levels of pro-inflammatory markers CRP ng/ml (A, B), sCD27 U/ml (C, D) and sCD30 pg/ml (E, F) were correlated with post-vaccination (day 30) OPT against tested serotype 23F in HIV-negative (left column) and HIV-positive (right column).
(TIF)

S3 Fig Serum levels of TNFs do not correlate with post-vaccination 23F opsonophagocytic titers (OPT). Serum levels of TNFs BAFF pg/ml (A, B), BCMA pg/ml (C, D) and TACI pg/ml (E, F) were correlated with post-vaccination (day 30) OPT against tested serotype 23F in HIV-negative (left column) and HIV-positive (right column).
(TIF)

S4 Fig PPS23F-specific naive B cells showed enhanced expression of TACI but not other receptors in HIV-positive and -negative individuals. Surface expression of BAFF-R (A), TACI (B), BCMA (C), CD40 (D) and CD21 (E), in naïve IgM and switched B cells were analyzed on PPS23F-selected B cells of HIV-positive (grey bars with slanted stripes) and HIV-negative (white bars with slanted stripes) individuals on day 7 and compared to day 0 unselected B cells in HIV-positive (plain grey bars) and HIV-negative (plain white bars) individuals. Data is represented as mean ± SEM. *p<0.05, **p<0.01, ***p<0.001.
(TIF)

S5 Fig HIV-viremic individuals show enhanced serum BAFF, sCD30 and CD21^lo^ B cells. Serum levels of BAFF pg/ml (A), sCD30 (B), surface expression of CD21 on unselected and PPS23F-selected memory B cells (C) and -naïve B cells (D) were evaluated in HIV-negative (white bars), HAART naïve, HIV-viremic (light grey) and HAART-experienced (dark grey) individuals. Data is represented as mean ± SEM. *p<0.05, **p<0.01, ***p<0.001.
(TIF)

S1 File Zipped folder containing TRENDS checklist and study protocol. **Trends Statement Checklist:** Trends Statement checklist for the study Inflammatory Markers and Immune Response to Pneumococcal vaccination in HIV-positive and -negative adults. **Study Protocol:** IRB Approved Study protocol for the assessment of Immune Response to Pneumococcal vaccination in HIV-positive adults.
(ZIP)
