# Supplementary figures and images for: Inflammatory Markers and Immune Response to Pneumococcal Vaccination in HIV-Positive and -Negative Adults
Source: PLoS One. 2016 Mar 1;11(3):e0150261. doi: 10.1371/journal.pone.0150261 (PMC4773189; doi:10.1371/journal.pone.0150261)

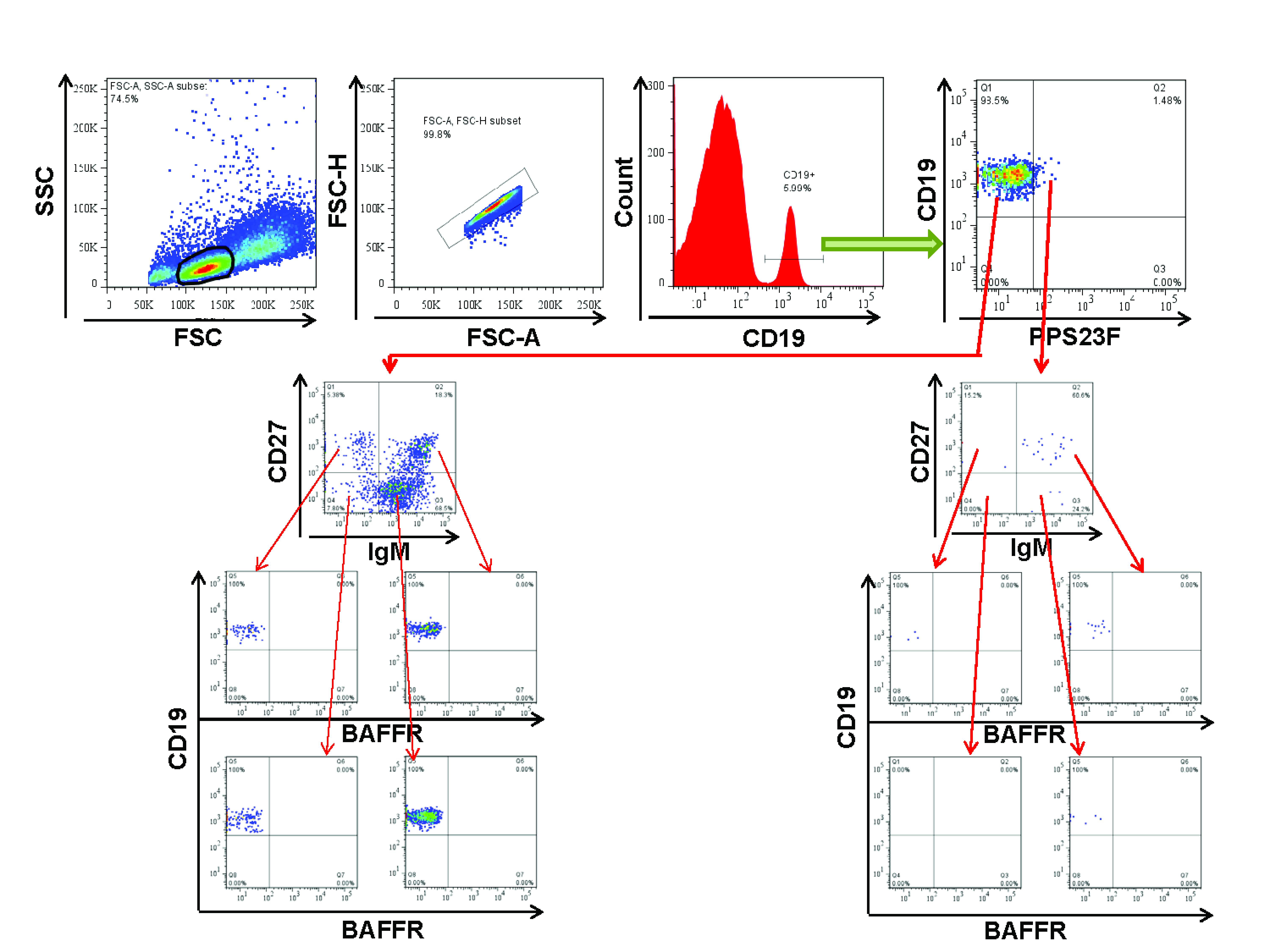

Supplement: S1 Fig — Lymphocytes were stained with fluorescently labeled PPS23F and antibodies for phenotypic characterization of cells. Lymphocytes were plotted (FSC-A, FSC-H) for doublet discrimination. Singlet lymphocytes were assessed for the expression of CD19+ B cells. B cells were plotted using 5-DTAF: PPS23F to identify PPS23F-selected vs. unselected cells. PPS-selected and unselected cells were further divided into sub-populations: naive (CD27−IgM+/-), class-switched memory (CD27+IgM−) and IgM memory (CD27+IgM+) B cells. Total (unselected), PPS23F-selected and respective memory and naïve B cell subsets were analyzed for the expression of BAFF-R as indicated. All flow cytometry results were plotted and analyzed using fluorescence minus one controls (FMO). 50,000 events were recorded. Data are represented as mean ± SEM. *p<0.05, **p<0.01, ***p<0.001. (TIF) [file pone.0150261.s001.tif]

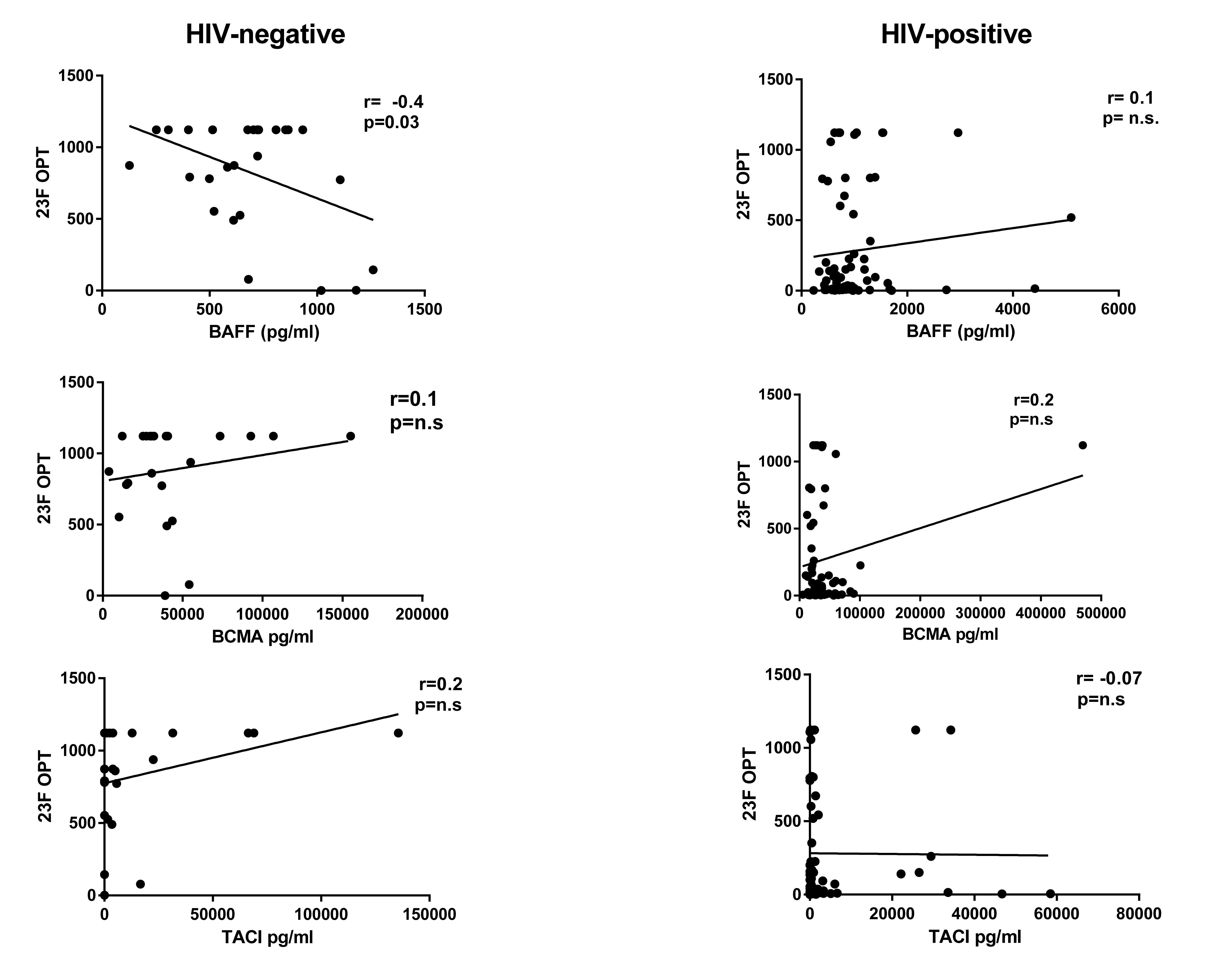

Supplement: S3 Fig — Serum levels of TNFs BAFF pg/ml (A, B), BCMA pg/ml (C, D) and TACI pg/ml (E, F) were correlated with post-vaccination (day 30) OPT against tested serotype 23F in HIV-negative (left column) and HIV-positive (right column). (TIF) [file pone.0150261.s003.tif]

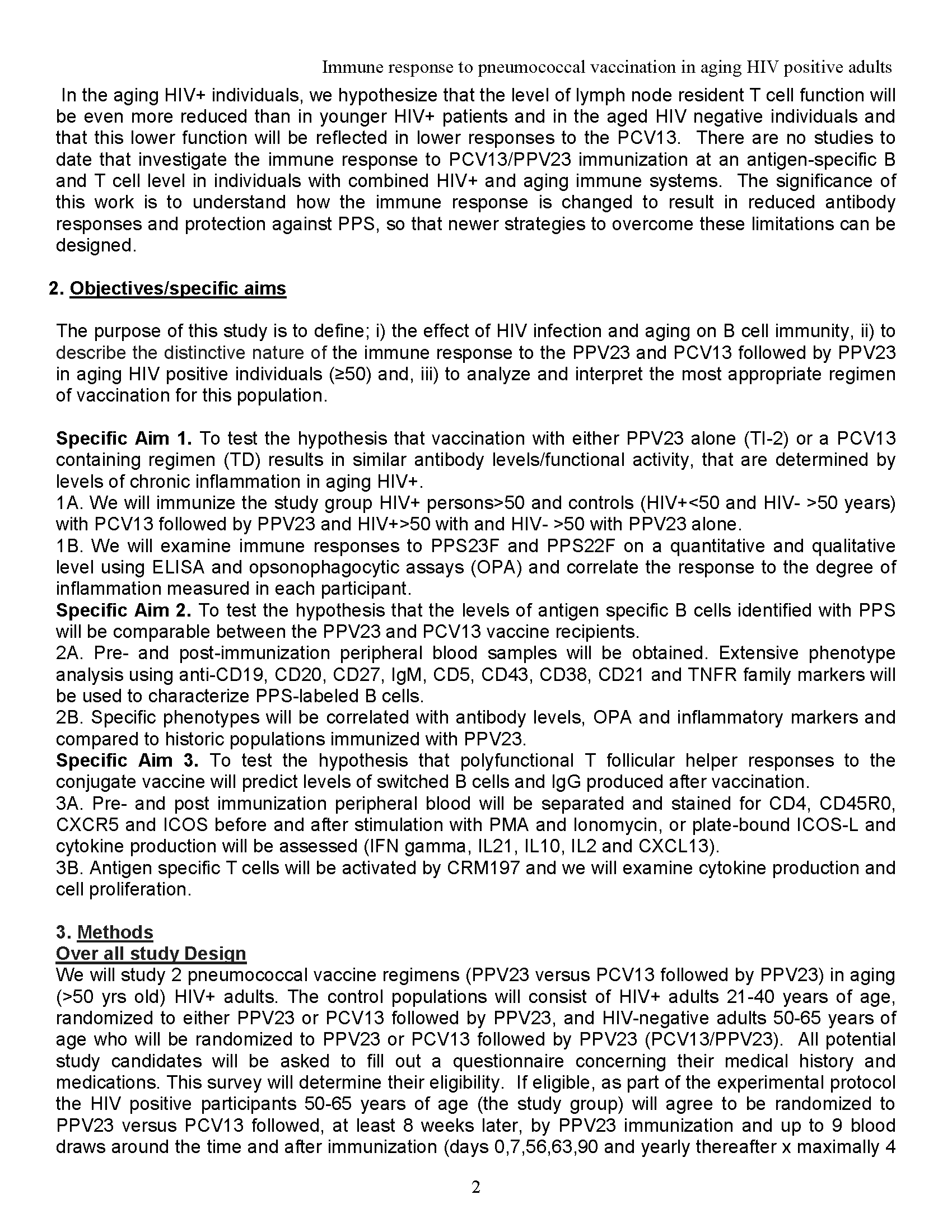

Supplement: S1 File — Trends Statement Checklist: Trends Statement checklist for the study Inflammatory Markers and Immune Response to Pneumococcal vaccination in HIV-positive and -negative adults. Study Protocol: IRB Approved Study protocol for the assessment of Immune Response to Pneumococcal vaccination in HIV-positive adults. (ZIP) [file pone.0150261.s006.zip › IRB Study Protocol_Page_2.tif]

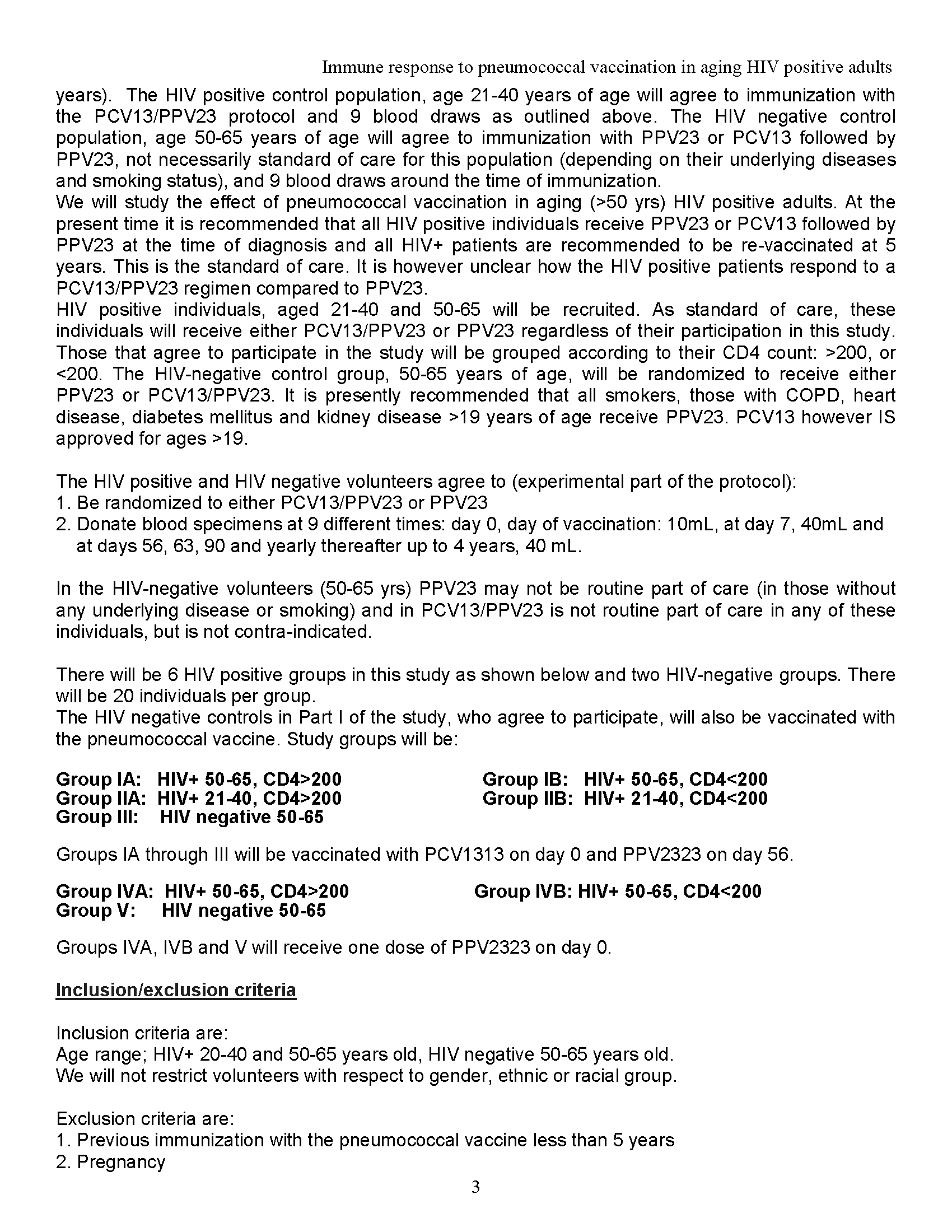

Supplement: S1 File — Trends Statement Checklist: Trends Statement checklist for the study Inflammatory Markers and Immune Response to Pneumococcal vaccination in HIV-positive and -negative adults. Study Protocol: IRB Approved Study protocol for the assessment of Immune Response to Pneumococcal vaccination in HIV-positive adults. (ZIP) [file pone.0150261.s006.zip › IRB Study Protocol_Page_3.tif]

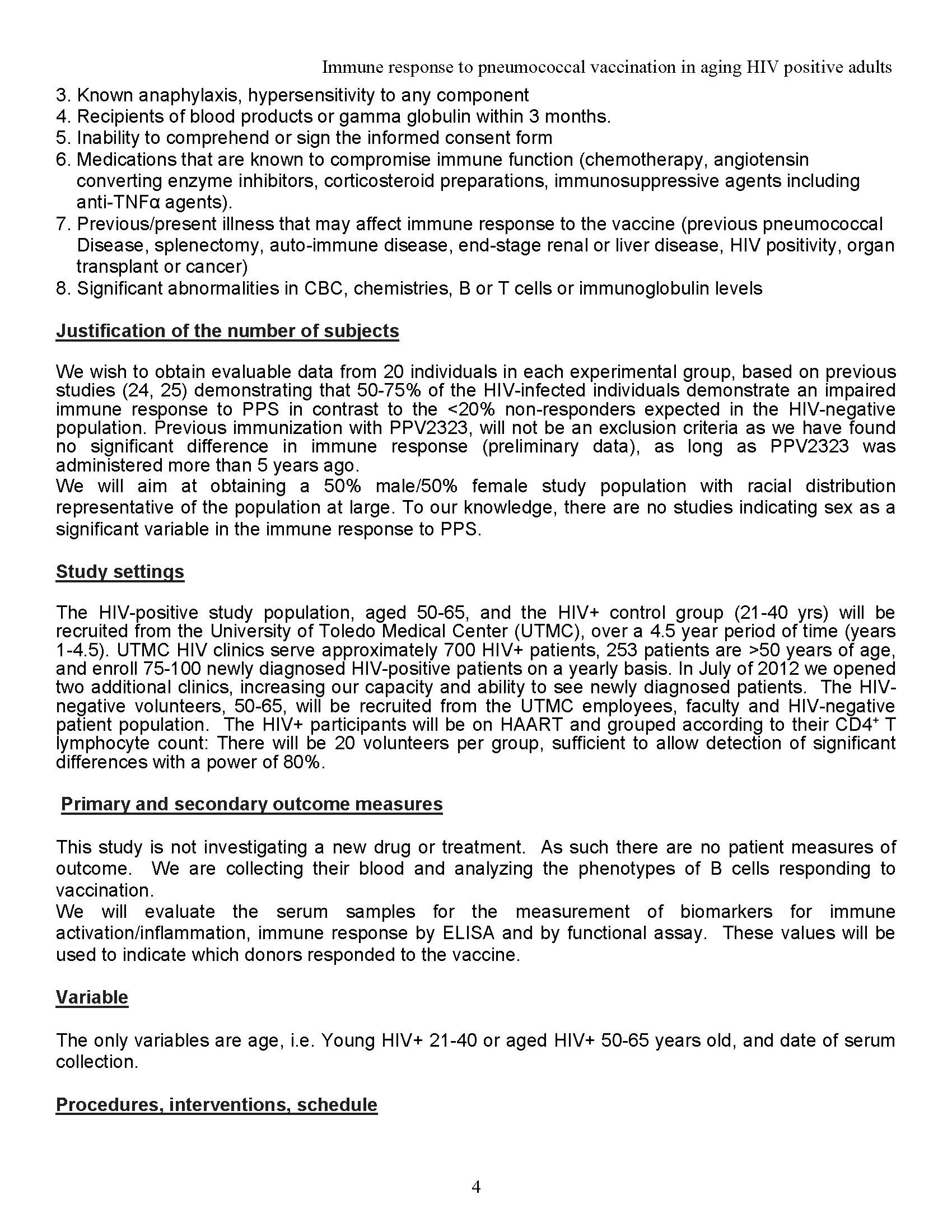

Supplement: S1 File — Trends Statement Checklist: Trends Statement checklist for the study Inflammatory Markers and Immune Response to Pneumococcal vaccination in HIV-positive and -negative adults. Study Protocol: IRB Approved Study protocol for the assessment of Immune Response to Pneumococcal vaccination in HIV-positive adults. (ZIP) [file pone.0150261.s006.zip › IRB Study Protocol_Page_4.tif]

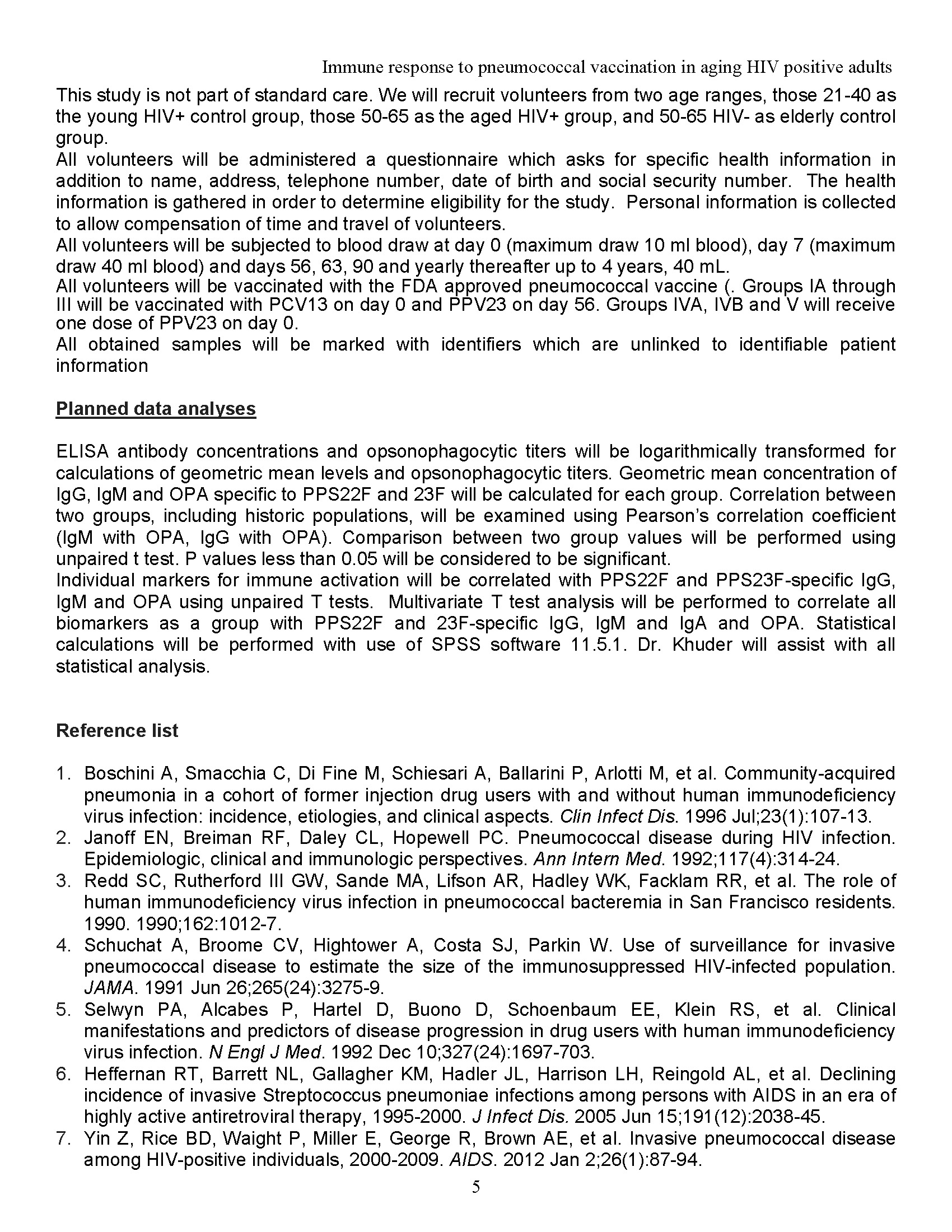

Supplement: S1 File — Trends Statement Checklist: Trends Statement checklist for the study Inflammatory Markers and Immune Response to Pneumococcal vaccination in HIV-positive and -negative adults. Study Protocol: IRB Approved Study protocol for the assessment of Immune Response to Pneumococcal vaccination in HIV-positive adults. (ZIP) [file pone.0150261.s006.zip › IRB Study Protocol_Page_5.tif]

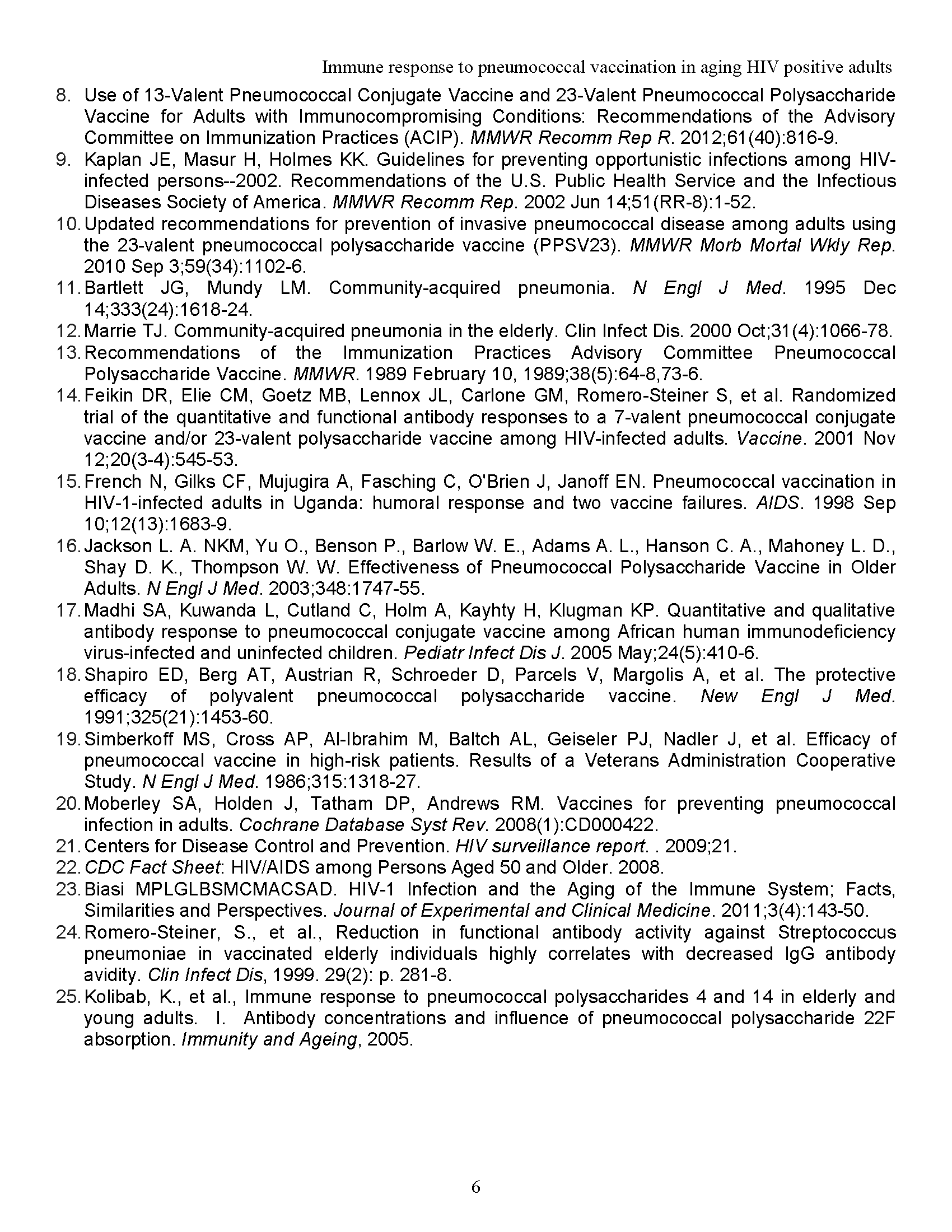

Supplement: S1 File — Trends Statement Checklist: Trends Statement checklist for the study Inflammatory Markers and Immune Response to Pneumococcal vaccination in HIV-positive and -negative adults. Study Protocol: IRB Approved Study protocol for the assessment of Immune Response to Pneumococcal vaccination in HIV-positive adults. (ZIP) [file pone.0150261.s006.zip › IRB Study Protocol_Page_6.tif]

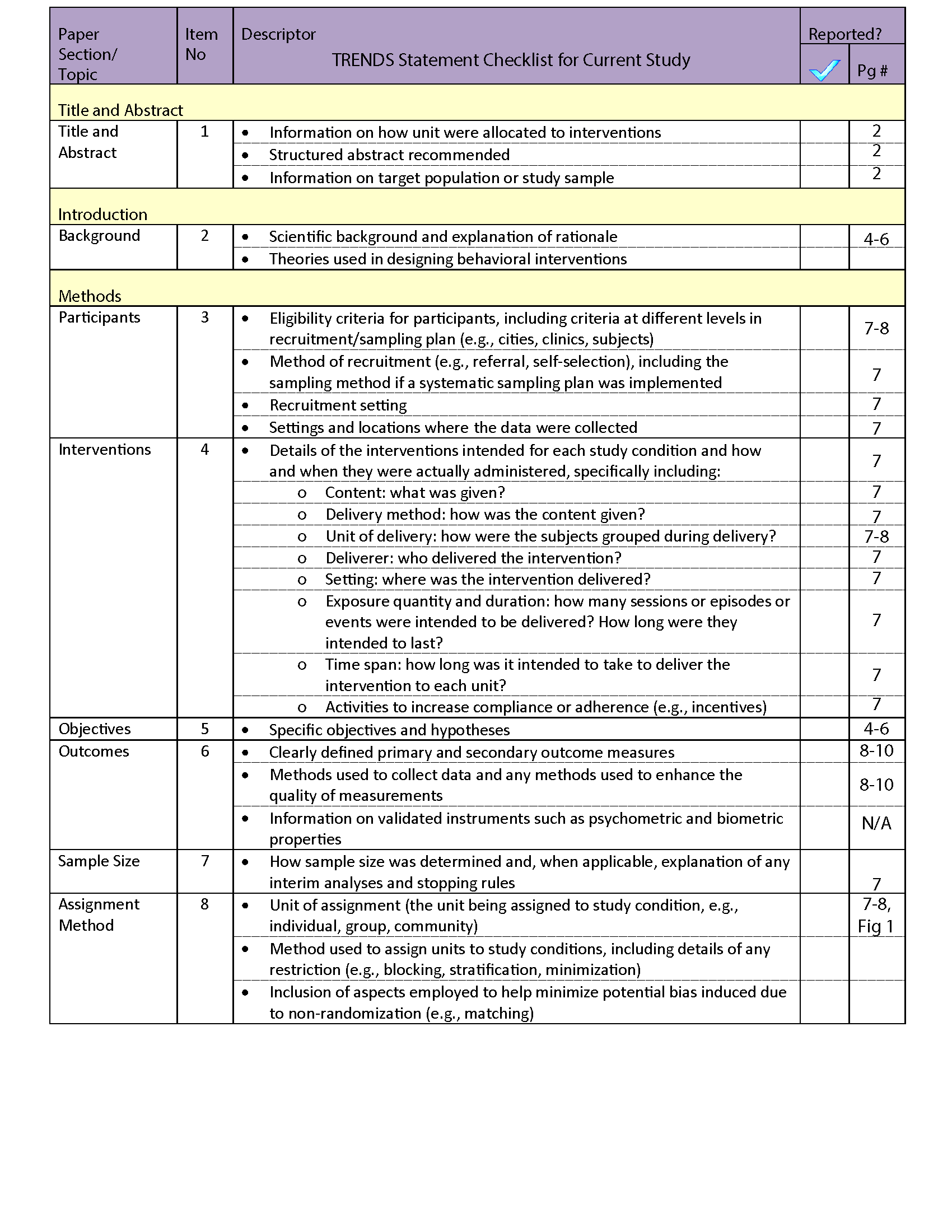

Supplement: S1 File — Trends Statement Checklist: Trends Statement checklist for the study Inflammatory Markers and Immune Response to Pneumococcal vaccination in HIV-positive and -negative adults. Study Protocol: IRB Approved Study protocol for the assessment of Immune Response to Pneumococcal vaccination in HIV-positive adults. (ZIP) [file pone.0150261.s006.zip › S Trends Checklist_Page_1.tif]

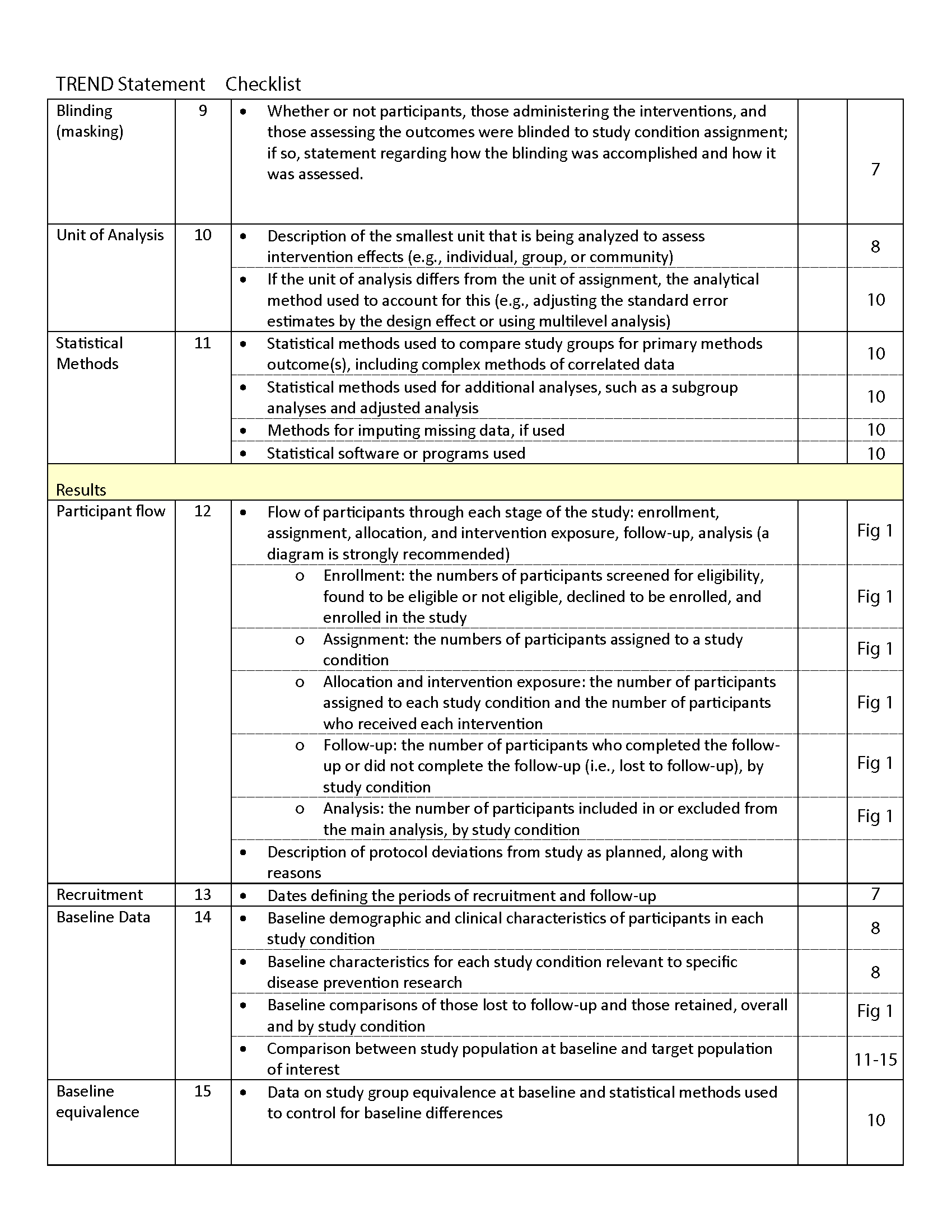

Supplement: S1 File — Trends Statement Checklist: Trends Statement checklist for the study Inflammatory Markers and Immune Response to Pneumococcal vaccination in HIV-positive and -negative adults. Study Protocol: IRB Approved Study protocol for the assessment of Immune Response to Pneumococcal vaccination in HIV-positive adults. (ZIP) [file pone.0150261.s006.zip › S Trends Checklist_Page_2.tif]

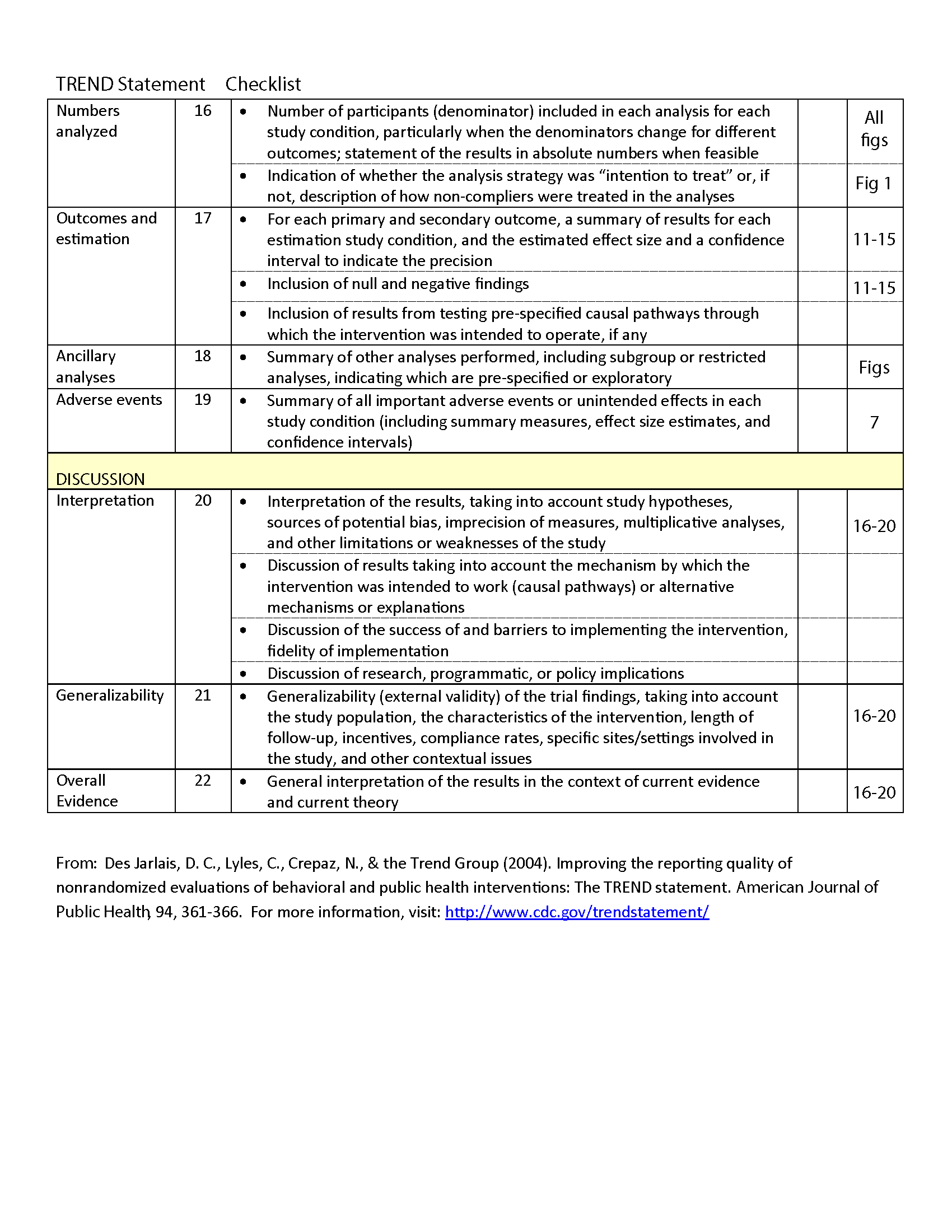

Supplement: S1 File — Trends Statement Checklist: Trends Statement checklist for the study Inflammatory Markers and Immune Response to Pneumococcal vaccination in HIV-positive and -negative adults. Study Protocol: IRB Approved Study protocol for the assessment of Immune Response to Pneumococcal vaccination in HIV-positive adults. (ZIP) [file pone.0150261.s006.zip › S Trends Checklist_Page_3.tif]
